# Genetic relationship of *Staphylococcus aureus* isolated from humans, animals, environment, and Dangke products in dairy farms of South Sulawesi Province, Indonesia

**DOI:** 10.14202/vetworld.2022.558-564

**Published:** 2022-03-10

**Authors:** Sartika Juwita, Agustin Indrawati, Retno Damajanti, Safika Safika, Ni Luh Putu Ika Mayasari

**Affiliations:** Department of Animal Disease and Veterinary Public Health, Faculty of Veterinary Medicine, IPB University, Bogor, Indonesia

**Keywords:** dairy farm, environment, human, *nuc* gene, *Staphylococcus aureus*

## Abstract

**Background and Aim:**

*Staphylococcus aureus* is a bacterium that causes several infectious diseases, including mastitis, endocarditis, and osteomyelitis, and poses a threat to human and animal health. This study aims to phenotypically and genetically identify *S. aureus* from the isolates collected from humans, animals, environment, and Dangke products in the dairy farms of South Sulawesi Province, Indonesia, as well as to establish a genetic relationship among the isolated *S. aureus* strains.

**Materials and Methods:**

The total number of samples was 142, comprising 30 humans (skin swab), 58 animals (raw milk), 14 dairy products (Dangke), and 40 environmental samples (water). *S. aureus* was phenotypically identified using the culture method, followed by Gram staining, catalase test, and coagulase test. Simultaneously, genotypic identification of *S. aureus* was performed using the conventional polymerase chain reaction and sequencing methods. Sequencing data were analyzed using the MEGA X software by comparing BLAST National Center for Biotechnology Information databases.

**Results:**

The phenotypic methods revealed that 56/142 (39.4%) animal, human, and Dangke samples grew on culture, and 56/56 (100%) were Gram stain positive, 56/56 (100%) catalase-positive, and 23/56 (41.1%) coagulase positive. The genotypic method revealed that 32/56 (57.1%) samples amplified the *nuc* gene. The phylogenetic analysis of 12 isolates revealed that they are all closely related and do not belong to distinct clades.

**Conclusion:**

It indicates that *S. aureus* isolates from animals (S30) are probably the same strain as human isolates (H2, H3, H4, and H5). The findings of this study can be used as information regarding the importance of preventing and controlling diseases caused by *S. aureus* using a health approach involving the human, animal, and environmental sectors. This study was limited to the sequencing analysis of the *nuc* gene.

## Introduction

*Staphylococcus aureus* is a bacterium that causes several infectious diseases and poses a threat to human and animal health. *S. aureus* is a type of bacteria that causes clinical and subclinical mastitis in dairy cattle. The infection by these bacteria can result in economic losses for the global dairy industry [1-3]. *S. aureus*, also known as commensal bacteria, colonizes the nostrils and skin in humans. It is also an opportunistic pathogen, causing superficial skin and soft-tissue infections, as well as potentially life-threatening invasive diseases, including bacteremia, pneumonia, endocarditis, and osteomyelitis [4]. *S. aureus* can also survive in the environment, including water, manure, or the air [5]. *S. aureus* can be transmitted through the interaction between individuals (directly or contaminated objects) [6]. In addition, *S. aureus* can be transmitted zoonotically by direct contact with animals or animal products [7]. Furthermore, *S. aureus* produces a thermostable extracellular nuclease (thermonuclease/TNase) encoded by the *nuc* gene, which is a virulence factor and one of the essential characteristics. Therefore, it can be used to differentiate *S. aureus* from *Staphylococcus* spp. The *nuc* gene is frequently used as a specific target for identifying *S. aureus* through polymerase chain reaction (PCR) [8,9].

Reliable identification of *S. aureus* is a significant concern in clinical microbiological diagnosis. Identifying *S. aureus* can use phenotypic and ­genotypic methods. Phenotypic methods include the culture method, followed by Gram staining, fermentation, catalase, and coagulase [10,11]. Genotypic methods include comparative methods based on standard band electrophoresis, genome characterization, and sequencing methods. Among the characterization methods, random amplified polymorphic DNA-PCR, repetitive element sequence-based PCR, restriction fragment length polymorphism-PCR, pulsed-filed gel electrophoresis-PCR, and multilocus sequence typing-PCR have been established as a rapid and straightforward technology for taxonomic and epidemiological analysis of numerous *Staphylococcus* species [12-15]. Rapid and precise identification of *S. aureus* from multiple potential sources of infection is crucial for disease prevention and control [16].

This study aims to phenotypically and genetically identify *S. aureus* from the isolates collected from humans, animals, environment, and Dangke products in the dairy farms of South Sulawesi Province, Indonesia, as well as establish a genetic relationship among the isolated *S. aureus* strains.

## Materials and Methods

### Ethical approval and informed consent

This study has received ethical approval from the Health Research Ethics Commission of Hasanuddin University Hospital (Number: 105/UN4.6.4.5.31/PP36/2021). Furthermore, all animal owners and human contacts gave their written informed consent to participate in this study before sampling.

### Study period and location

The study was conducted from March to June 2021 at Hasanuddin University Medical Research Centre and Disease Investigation Centre, Maros.

### Sample collection

The total number of samples was 142, comprising 30 humans (skin swab), 58 animals (raw milk), 14 dairy products (Dangke), and 40 environmental samples (water) obtained from 20 dairy farms (location A-T) in Enrekang Regency, South Sulawesi Province, Indonesia. Raw milk and water samples were collected in sterile tubes, Dangke was transferred to sterile bags, while skin swabs used Amies transport media (Oxoid Ltd., Hampshire, UK). All samples were transported to the laboratory at 4°C for bacterial analysis. The samples were analyzed in March and stored for 18 h before the analysis.

### Phenotypic methods

Ten milliliters of milk sample and 10 g of Dangke were inoculated into 90 mL of peptone water (PW; pH 7.2±0.2 at 25°C) broth (Oxoid Ltd.) and incubated at 37°C for 24 h. Next, samples grown on PW broth were spread onto Baird-Parker agar (BPA) (Oxoid Ltd.) and set at 37°C for 24-48 h [17]. Skin swab samples were inoculated directly on BPA media and incubated at 37°C for 24-48 h [18]. In addition, Gram staining, catalase test, and coagulase test were performed on bacterial colonies growing on BPA [19]. *S. aureus* ATCC 25923 (Dartford, UK) was used as a positive control.

### DNA extraction

The cultured bacterial colonies were extracted using the boiling or heating method described by Hassanzadeh *et al*. [20], with modifications. First, one loop of bacterial colonies was transferred into 500 μL of Tris-EDTA buffer (Invitrogen, USA) and vortexed. Next, 200 μL of bacterial cell suspension was heated at 95°C for 25-30 min before being centrifuged at 16060 x g for 15 min. Then, the resulting pellet was added to 200 μL of nuclease-free water and then homogenized.

### Genotypic methods

Bacterial colonies were grown on BPA followed by a conventional PCR to identify *S. aureus*. Confirmation of *S. aureus* used the *nuc* gene target. Primer design was forward 5′-GCGATTGATGGTGATACGGTT-3′ and primer reverse 5′-AGCCAAGCCTT GACGAACTAAAGC-3′ [21]. The product PCR exhibits a size of 270 bp. DNA amplification was performed using a PCR Machine (T100^™^ Thermal Cycler; Bio-Rad, California, USA). The total volume of PCR reaction was 25 μL, including 5 μL DNA, 0.5 μL of forward primer and reverse primer, 6.5 μL nuclease-free water, and 12.5 μL of KAPA2G Fast Ready Mix PCR Kit (KAPA Biosystems, Cape Town, South Africa) used in each reaction. The PCR conditions used were as follows: Pre-denaturation at 95°C for 5 min, followed by 35 cycles of denaturation at 95°C for 15 s, annealing at 55°C for 15 s, and extension at 72°C for 30 s, followed by a final extension at 72°C for 5 min. A total of 5 μL were visualized with 2% agarose gel with 1 μL Gel Red® Nucleic Acid Stain (Biotium Inc., California, USA). Then, the PCR products were sequenced at 1^st^ Base in Selangor, Malaysia. Several sample isolates were selected as representatives for sequencing.

### Statistical analysis

The *nuc* gene sequencing results were read using sequence scanner v1.0 software (Applied Biosystem, USA), and the data were saved in FASTA format. Then, the data were analyzed using MEGA X (https://www.megasoftware.net). Next, data were submitted to the BLAST process at National Center for Biotechnology Information database to identify isolates (https://www.ncbi.nlm.nih.gov/). Finally, the phylogenetic tree was reconstructed. The isolates were identified based on the highest homology percentages to the reported sequences.

## Results

**Figure-1:**
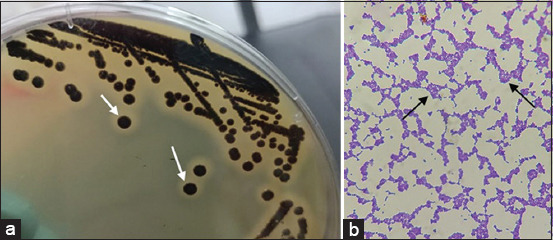
*Staphylococcus aureus* colonies (white arrows) on Baird-Parker agar (a) and *Staphylococcus* *aureus* cell morphology (black arrows) at 1000× (b).

The results of phenotypic methods showed that 56 (39.4%) samples from 142 samples grew on BPA. *S. aureus* colonies on BPA were round, smooth, convex, 2-3 mm in diameter, gray to black, and had clear colony edges (halo-formed) (Figure-1). Samples of Dangke, an unfermented dairy product, showed positive growth of bacteria of 35.7%. In addition, it was revealed that the isolated *S. aureus* strains were 100% gram-positive, 100% catalase-positive, and 41.1% coagulase-positive (Table-1).

**Table-1:** The results of the phenotypic and genotypic methods.

Samples	Number of samples	Phenotypic methods				Genotypic method
	
		Culture (%)	Gram staining (%)	Catalase test (%)	Coagulase test (%)	*nuc* gene target (%)
Animal* Human**	58	32/58 (55.2)	32/32 (100)	32/32 (100)	10/32 (31.2)	20/32 (62.5)
Farmer	20	15/20 (75)	15/15 (100)	15/15 (100)	7/15 (46.7)	7/15 (46.7)
Dangke maker	10	4/10 (40)	4/4 (100)	4/4 (100)	2/4 (50)	1/4 (25)
Dangke	14	5/14 (35.7)	5/5 (100)	5/5 (100)	4/5 (80)	4/5 (80)
Environment						
Water sources	20	0	0	0	0	0
Animal waste	20	0	0	0	0	0
Total	142	56/142 (39.4)	56/56 (100)	56/56 (100)	23/56 (41.1)	32/56 (57.1)

* Raw milk samples

** Skin swab sample

**Figure-2:**
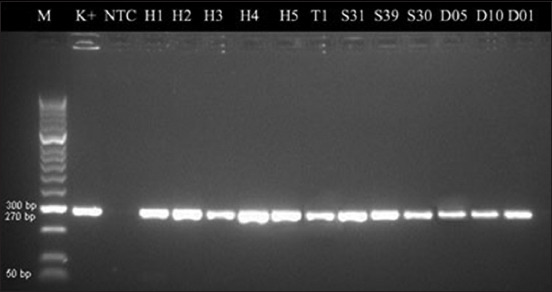
Results of *nuc* gene amplification (270 bp). M: Marker 50 bp; positive controls: ATCC 25923; sample code: Human (H1, H2, H3, H4, H5, and T1), animal (S31, S39, and S30), and Dangke (D05, D10, and D01); NTC=Non-template control.

**Figure-3:**
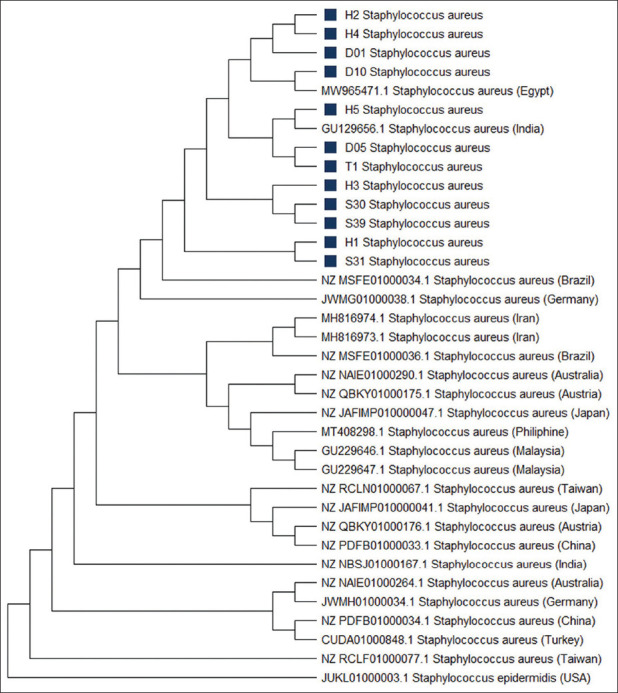
Phylogenetic tree construction. Isolates used in this research are shown in the black box. Phylogenetic analysis was constructed based on the neighbor-joining tree with a two-parameter Kimura substitution model. *Staphylococcus epidermidis* as an out-group.

In this study, the genotypic method revealed that 32/56 (57.1%) samples amplified the *nuc* gene by PCR (Table-1). Figure-2 depicts the result of PCR amplification of the *nuc* gene. Furthermore, the phylogenetic tree construction (Figure-3) showed that isolates of humans (H1, H2, H3, H4, H5, and T1), animal (S31, S39, and S30), Dangke (D05, D10, and D01) are in the same group of strains of *S. aureus*. Isolates of H1 and S31 came from the exact farm (location A), and isolates T1 and D01 also originated from the exact farm (location T), while the other isolates came from different farms. The isolates from dairy farms in South Sulawesi Province were genetically closely associated with *S. aureus* isolates from India (GU129656.1) and Egypt (MW965471.1). The genetic distances of the 12 *S. aureus* isolates differed (Table-2), with the closest genetic distance being 0.00 and the farthest distance being 0.02.

**Table-2:** The genetic distance among *Staphylococcus aureus* isolates.

Number of samples	Number of samples											

	1	2	3	4	5	6	7	8	9	10	11	12
1. S31		0.01	0.01	0.01	0.01	0.01	0.01	0.01	0.01	0.01	0.01	0.01
2. S39	0.01		0.01	0.01	0.01	0.01	0.01	0.01	0.01	0.01	0.01	0.01
3. S30	0.01	0.01		0.01	0.00	0.00	0.00	0.00	0.01	0.00	0.00	0.01
4. H1	0.01	0.02	0.02		0.01	0.01	0.01	0.01	0.01	0.01	0.01	0.01
5. H2	0.01	0.01	0.00	0.02		0.00	0.00	0.00	0.01	0.00	0.00	0.01
6. H3	0.01	0.01	0.00	0.02	0.00		0.00	0.00	0.01	0.00	0.00	0.01
7. H4	0.01	0.01	0.00	0.02	0.00	0.00		0.00	0.01	0.00	0.00	0.01
8. H5	0.01	0.01	0.00	0.02	0.00	0.00	0.00		0.01	0.00	0.00	0.01
9. T1	0.01	0.01	0.01	0.02	0.01	0.01	0.01	0.01		0.01	0.01	0.01
10. D05	0.01	0.01	0.00	0.02	0.00	0.00	0.00	0.00	0.01		0.00	0.01
11. D01	0.01	0.01	0.00	0.02	0.00	0.00	0.00	0.00	0.01	0.00		0.01
12. D10	0.01	0.01	0.00	0.02	0.01	0.01	0.01	0.01	0.01	0.01	0.01	

## Discussion

This study aims to phenotypically and genetically identify *S. aureus* from the isolates collected from humans, animals, environment, and Dangke products in the dairy farms of South Sulawesi Province, Indonesia, as well as to establish a genetic relationship among the isolated *S. aureus* strains.

The results showed the growth of *S. aureus* from the sample of animals (raw milk), humans (skin swabs), and Dangke. Meanwhile, no *S. aureus* was documented from the environmental sample. In this study, the prevalence of *S. aureus* in raw milk was higher than the results of the previous studies [22,23]. *S. aureus* contaminates many raw milk sources commonly associated with mastitis or human carriers [24]. The growth of *S. aureus* in dairy products (Dangke) indicates contamination, which is probably the result of the unhygienic handling of the product. Improper food handling practices contribute to bacterial contamination of products [25]. In this study, *S. aureus* was found colonized in the skin of 75% of dairy farmers and 40% of Dangke makers, which is higher than the previous study, which reported colonization in 25% of dairy farmers [26]. *S. aureus* cross-transmission between humans and animals is frequently documented between farmworkers and dairy cattle due to their proximity [27]. During milking, the hands of farmers appear to be the principal transmitters of *S. aureus* to the dairy cow[24].

*S. aureus* produces the enzyme catalase, which functions in cells to prevent hazardous level of hydrogen peroxide (H_2_O_2_) from accumulating as a by-product of metabolic activities, especially the electron transport pathway [28]. Catalase is a heme protein enzyme that decomposes H_2_O_2_ into water and oxygen. This reaction is evidenced by the rapid formation of air bubbles [29]. In the case of *S. aureus* strains that tested negative for catalase, mutations in the *katA* gene (catalase gene) were discovered [30].

*S. aureus* produces coagulase enzyme is a polypeptide that binds and activates prothrombin, so that it will convert fibrinogen into fibrin and increase plasma or blood clotting [31]. In this study, 42% of the isolates were coagulase-positive and 58% were coagulase-negative. Coagulase-negative *S. aureus* is also found in raw milk samples and clinical specimens [32,33]. In addition, a study reported a coagulase-negative mutant of *S. aureus* [34].

In the past, the existence of the *nuc* gene was used to identify *S. aureus*. The *nuc* gene is discovered in most *S. aureus* isolates; nevertheless, certain isolates have been discovered that lack this gene. The previous report identified clinical and raw milk samples from dairy as positive for the *nuc* gene of 84% and 100%, respectively [33,35]. The chromosome of *S. aureus* encodes thermonucleases, *nuc* gene. The *nuc* gene is known as a specific virulence factor in *S. aureus* [36], and it contributes to biofilm formation [37] and immune evasion [38].

Differences are found between the results of the phenotypic and genotypic methods for detecting *S. aureus* strains. When using phenotypic approaches, false positives are common, especially when dealing with identical microbial species. Furthermore, phenotypic methods cannot identify microorganisms to the species level, let alone the strain level, and cannot detect cells that cannot be cultivated. However, genotypic approaches aided metagenomic investigations of vast and diverse bacterial communities by allowing identification of previously unknown species, characterization of non-cultured bacteria, and metagenomic studies of previously unknown taxa [39-41].

A phylogenetic tree was developed to evaluate the genetic relationship between the locally identified *S. aureus* and reported *S. aureus* based on the *nuc* gene sequences. The result indicates that the sample isolates belonged to a monophyletic group with other *S. aureus* strains. Based on genetic distance, animal isolate (S30) exhibits the most relative distance to humans (H2, H3, H4, and H5), indicating that *S. aureus* isolates from animals are probably the same strain as human isolates. The strain of adapting after a long-term relationship with humans is expected to cause a host jump from humans to animals. Close contact between humans and animals can facilitate host-switching occurrences [42]. Domesticated animal studies revealed that a host switch from humans occurred in the past [43]. The ability of some pathogens to transfer from one host species to another poses a significant hazard to public health and food security [44].

## Conclusion

Phenotypic methods detected *S. aureus* in 39.4% samples of animal, human, and Dangke, while genotypic methods identified 57.1% of samples as positive for *S. aureus*. Thus, the phylogenetic analysis of the 12 isolates showed that they were closely related and did not belong to distinct clades. The findings of this study can be used as information regarding the importance of preventing and controlling diseases caused by *S. aureus* using a health approach involving the human, animal, and environmental sectors.

## Authors’ Contributions

SJ: Designed the study, collected the data, interpreted the data, and drafted the manuscript. AI, RD, SS, and NLPIM: Designed the study, analyzed the data, and drafted and revised the manuscript. All authors read and approved the final manuscript.
